# COVID-19: Impact on United Kingdom Horse Owners

**DOI:** 10.3390/ani10101862

**Published:** 2020-10-13

**Authors:** Jane M. Williams, Hayley Randle, David Marlin

**Affiliations:** 1 Hartpury University, Gloucester GL19 3BE, UK; jane.williams@hartpury.ac.uk; 2 School of Animal and Veterinary Sciences, Wagga Wagga Campus, Charles Sturt University, North Wagga Wagga NSW 2650, Australia; hrandle@csu.edu.au; 3 David Marlin Consulting, P.O. BOX 187, Cambridge CB4 0WZ, UK

**Keywords:** coronavirus, equine welfare, equine management, horse–human relationship

## Abstract

**Simple Summary:**

On 11 March 2020, the World Health Organization declared coronavirus disease (COVID-19) outbreak a global pandemic and an ongoing public health emergency. In the United Kingdom, quarantine and social distancing measures were implemented with immediate effect on 17 March 2020, resulting in a rapid change to the way owners managed and interacted with their horses. We surveyed 6259 horse owners to evaluate the impact of COVID-19 on themselves and their horses. The majority of horse owners were visiting and riding their horses less, with increased restrictions experienced by owners who kept their horse at private livery yards. Whilst social distancing and visiting restrictions were in place at livery yards, nearly half were not providing hand sanitization or disinfection protocols for shared areas/equipment to prevent the spread of the virus between owners. Horse owners expressed concern that equine health and welfare would be negatively affected by the restrictions put in place and of financial consequences as a result of the pandemic. The majority of respondents also felt their own mental health and wellbeing was being adversely affected by not being able to visit/interact as they normally would with their horses. Equestrian influencers and national bodies should engage with increased communication and education to support horse owners through the pandemic in the short, medium, and long-term.

**Abstract:**

COVID-19 was declared a global pandemic on 11 March 2020; the United Kingdom (UK) implemented quarantine measures shortly afterward, resulting in rapid changes in how owners managed and interacted with their horses. This study provides a rapid analysis of the initial impact of the COVID-19 outbreak on the management of UK leisure and competition horses. A 17 question online survey was distributed via equestrian social media sites to ascertain the impact of COVID-19 on horse and yard management and on human–horse interactions. Frequency analysis combined with Chi-squared and thematic analyses identified the impact of COVID-19 on UK horse owners. Major changes within horse management and horse–human interactions were reported for the majority of horse owners (>65%), regardless of the establishment type or region. Social distancing and visiting restrictions were implemented at most yards, but nearly half were not providing hand sanitization or disinfection protocols for the shared areas/equipment to prevent cross-contamination between users. The financial impact of the pandemic combined with restricted access to veterinary professionals resulted in owners expressing concerns that horse health and welfare may be compromised as a result. Horse owners also felt that the reduced opportunities for horse–human interactions were negatively affecting their mental health and wellbeing.

## 1. Introduction

Coronaviruses are a complex of RNA viruses that are highly pathogenic, comprise four genera, and that have crossed species barriers for over a thousand years, enabling them to affect a diverse range of hosts, with seven strains identified as important human pathogens that primarily target the human respiratory system [1]. Recent coronaviruses that have been associated with previous global pandemics include severe acute respiratory syndrome (SARS)-CoV and Middle East respiratory syndrome (MERS)-CoV. In December 2019, initial accounts of a pneumonia outbreak of unknown cause were reported in Wuhan, Hubei Province, China [2,3]. These were later associated with a novel coronavirus, the Severe Acute Respiratory Syndrome coronavirus 2 (SARS-COV-2), subsequently named coronavirus COVID-19 by the World Health Organisation (WHO) in February 2020. At the same time, the WHO predicted the potential of a COVID-19 global pandemic [1,2].

**Figure 1:**
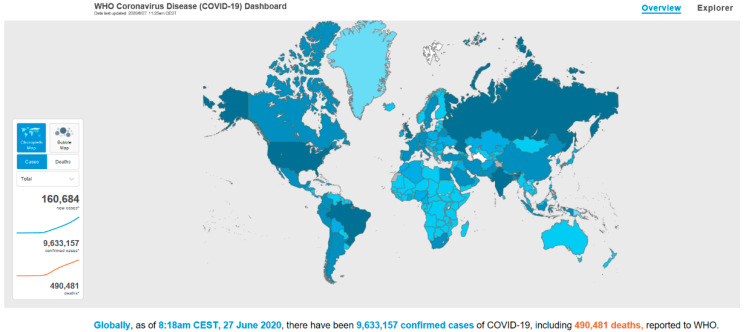
The World Health Organisation corona virus (COVID-19) dashboard 27 June 2020.

On 11 March 2020, the WHO declared COVID-19 a global pandemic and an ongoing public health emergency [4,5]. At the time of preparing this manuscript (27 June 2020), 9,633,157 cases of SARS-CoV-2 globally, across 215 countries with 490,481 deaths, were recorded by the WHO (https://www.who.int/emergencies/diseases/novel-coronavirus-2019) (Figure 1). The first SARS-CoV-2 cases occurred in the United Kingdom (UK) in late January 2020, with the first death recorded on the 5th of March. As of the 26th of June 2020 a total of 309,360 positive cases and 43,414 deaths occurred in the UK (https://www.gov.uk/guidance/coronavirus-COVID-19-information-for-the-public). Cases have been reported across all UK regions.

The aetiopathogenesis of SARS-CoV-2 is not fully understood. Previous epidemiological studies highlighted three factors involved in viral spreading: the source of infection, the route of transmission, and susceptibility [5,6]. The transmission rate of SARS-CoV-2 appears to be determined by the volume of interactions an infected person has with other people and the environment. The virus is reported to have a reproduction number (R0) ranging between 2.24 and 3.58 [2]. Close contact with a person infected with SARS-CoV-2 virus and environmental contamination from droplets spread by coughing or sneezing from an infected individual are the most common routes of transmission [1,2,5].

In addition, researchers have detected SARS-CoV-2 virus in samples of stool, the gastrointestinal tract, saliva, and urine in infected patients [5]. No studies to date have fully elucidated any potential natural hosts or intermediate hosts for SARS-CoV-2. Bats are considered likely to be natural hosts and pangolins are thought to be intermediate hosts [5]. The structural and biochemical properties of the SARS-CoV-2 virus suggests an affinity to angiotensin-converting enzyme 2 receptors on host cells, which could facilitate cross-species transmission, leading to many animals, including potentially horses, acting as intermediate hosts for the virus [6,7]. Transmission from humans to dogs, domestic cats, tigers, and lions has been reported, suggesting that these animals could act as vectors for the disease [8,9]; however, no cases of transmission from equid species have been confirmed at the time of writing.

While COVID-19 continues to spread across the globe, due to the risk of person to person transmission, the quarantine of individuals demonstrating COVID-19 symptoms or who have tested positive for the SARS-CoV-2 virus has been advised by the WHO to reduce transmission rates [2]. Enhanced measures, including extended isolation periods, are recommended in populations with the potential to become super-spreaders, including children and health care providers, and for susceptible groups, such as those with existing respiratory and immunological health issues, and elderly people [2,10]. On the 17 March 2020, the UK government implemented social distancing, requiring individuals and families to avoid contact with other people outside of their household as much as possible, alongside quarantine measures for households where a member had symptoms of SARS-CoV-2 and groups at high risk [11]. These measures were escalated to a nationwide lockdown on March 23rd 2020, with individuals advised to only go outside for food, health reasons, or work but only if working from home was not possible [12]. At the time of writing (27 June 2020), quarantine has been relaxed; however, social distancing measures are still in place in the UK.

The government responses to mitigate the spread of infection and to reduce mortality rates have rapidly altered daily lives and led to changes in how we engage in outdoor activities, such as horse riding [5,13]. The current COVID-19 pandemic is having a significant impact on the British equestrian sector [14]. The British Equine Veterinary Association has advised its members to provide essential emergency veterinary care only and services, such as farriery and equine dentistry, are subject to strict social distancing and infection control measures [15,16,17]. British Equestrian, the national governing body for horse sports in the UK, issued guidance for horse owners, including confirming that visiting your horse does qualify as an essential activity if you are their carer, and recommending that people should not ride their horse unless strictly necessary for welfare reasons to avoid placing unnecessary pressure on the National Health Service and emergency services [14]. General recommendations to reduce the spread of COVID-19 also apply to equestrian establishments, these currently include but are not limited to: staying 2 metres (6 ft) away from other people at all times, providing decontaminating reagents and facilities for cleaning hands on a routine basis, and not meeting others, even friends or family [12].

Equestrian sports and non-competitive leisure riding are popular in the UK, with approximately 3 million people regularly riding [18,19]. In the UK, horse sports and related activities provide a significant contribution (£4.7 billion in 2019) to the UK economy [19]. Horse owners and livery yard owners are responsible for the management of the horses under their care and should engage in practices that optimise equine health and welfare [20]. Equestrian practice is multifaceted and to achieve optimum management due consideration of the appropriate nutrition, housing, expression of normal behaviour, including socialisation, and the application of ethical riding and training practices, as well as ensuring the horse is sufficiently prepared in terms of fitness and skills development to meet the demands of the workload expected, are required [18,20,21].

Horses in the UK are managed in a variety of different ways from being based at home with their owners/professional trainers or riders, owners caring for horses at do-it-yourself (DIY) facilities, horses on part livery: a mixed model where owners manage some aspects/days of the horses’ daily care and pay for the facility to undertake other aspects, or full livery: where the owner pays for the horses’ care to be provided by professional staff, often including provision of daily exercise/riding. Management practices and facilities also vary across establishments, with some horses living exclusively outdoors and others stabled with restricted turnout, some premises having riding arenas and hacking onsite, and others having no or limited onsite exercise facilities available.

Horse owners also often extol the beneficial impact of physical contact with their horse and how their relationship with their horse/s promotes positive mental wellbeing and physical health [22,23,24]. The rapid implementation of changes to normal equine management practices and equestrian activities associated with the quarantine measures to control the impact of COVID-19 in the UK, therefore, have the potential to affect human and equine welfare, and warrant further investigation to develop strategies to lessen the impact of the pandemic.

While coronavirus disease (SARS-CoV-2 virus) continues to spread across the globe, with no vaccine and limited medical capacity to treat the disease, non-pharmaceutical interventions will remain the primary strategy to contain the pandemic [25]. Government enforced restrictions are likely to remain in place for up to 18 months, until a safe and stable SARS-CoV-2 vaccine is available [5]. These are likely to incorporate community mitigation strategies reducing both mass gatherings with super-spreader potential, but also community and social level events [26,27]. Whilst the short-term cost of containment is widely considered to be far lower than the long-term cost of non-containment [28], evaluation of the short and long term impacts of the pandemic on animal welfare is required. This study aimed to provide a rapid analysis of the initial impact of the SARS-CoV-2 coronavirus disease outbreak (COVID-19) and subsequent government restrictions on the management of domestic/leisure and competition horses within the United Kingdom.

## 2. Materials and Methods

### 2.1. Participants

Participants were recruited online via the sharing of a link to the survey on selected UK equine-related or discipline-specific social media (Facebook^®^ and Twitter^®^) groups including but not limited to British Dressage, Eventing UK, Horse & Hound, Horsetalk NZ, and Horsesport. To be eligible to participate, respondents were required to be over 18 years of age, a current horse owner and reside in the UK. The survey was anonymous and no personal data were collected. In order to reduce bias, the survey was promoted and disseminated by an independent third party media company (Fox Red Media, Crown House, Loughton IG10 4LF).

### 2.2. Survey Design

The survey was designed as an online questionnaire (Survey Monkey^®^, San Mateo, CA, USA) with 16 closed questions and 1 open free text question (Supplementary File S1). A draft survey was tested by 20 experienced users and edited to correct any errors before being fully deployed. The survey was live from 26 March 2020 for 10 days and 94% of the responses were obtained within the first 6 days. Ethical approval for the study was granted by the Hartpury University Ethics Committee.

Demographic factors: The respondents were asked questions relating to which region in the UK they lived, how many horses they owned, and if their horse/s had any existing or current medical conditions. They were also asked to state how they kept their horse: at home, DIY, or part or full livery.

*The impact of COVID-19 on horse management*: The respondents were asked if how they managed their horse had changed as a result of the COVID-19 pandemic, if any changes made were voluntary or enforced, whether they had been told or advised to ‘rough-off’: the process of preparing a fit/in-work horse to be turned out for a period of reduced exercise or rest/holiday usually at pasture, their horse and if they had experienced changes to professional services, including but not limited to veterinarians, farriers, equine dentists, and physiotherapists.

*The impact of COVID-19 on yard/facility management*: The respondents were asked if social distancing measures had been implemented on their yard and to identify which other control measures to prevent SARS-CoV-2 transmission had been put in place.

*The impact of COVID-19 on respondent interactions with their horse*: The respondents were asked whether they had reduced the number of times a day they visited their horse, whether they were still riding or not, and if they felt the pandemic would result in their horse having a period of prolonged stable confinement.

The respondents were also asked what affect they felt any ongoing financial implications of the coronavirus pandemic could have on how they could provide for their horse’s key needs e.g., livery costs, feed, forage, farrier bills, and veterinary bills.

### 2.3. Data Analysis

The data were exported from Survey Monkey™ to Microsoft Excel™ Version 2010 (Redmond, WA, USA). The data were grouped according to the horses’ management system type: home, DIY, or part or full livery and by their UK geographical region, to enable the impact of COVID-19 to be evaluated by these characteristics. Frequency analysis identified how the coronavirus pandemic had affected horse management, including access to equine professionals and riding, yard/facility management, and horse owner interactions with their horse.

The data were non-parametric and categorical; therefore, a series of Chi-squared analyses identified if regional differences or how horses were kept influenced the frequency of the participant responses. The significance was set at *p* < 0.05.

The respondents were also asked an open question to ascertain any further comments they had related to the impact of COVID-19 on horses and horse owners. Inductive content analysis of responses was undertaken utilizing tags (‘open-coding’) to create emergent themes (‘focused coding’) using a grounded theory approach [29].

## 3. Results

The BETA 2019 survey identified 374,000 horse owning households in Britain. A total of 6259 respondents took part in this survey resulting in a margin of error of ±1% at a 95% confidence interval (https://www.surveymonkey.com/mp/margin-of-error-calculator/). The majority of these owned one or two horses (66.8%; *n* = 4127), which were kept predominately away from home at a private livery yard or on grass livery in a private field (72.9%; *n* = 4468).

### 3.1. Impact of COVID-19 by Type of Establishment

**Figure 2:**
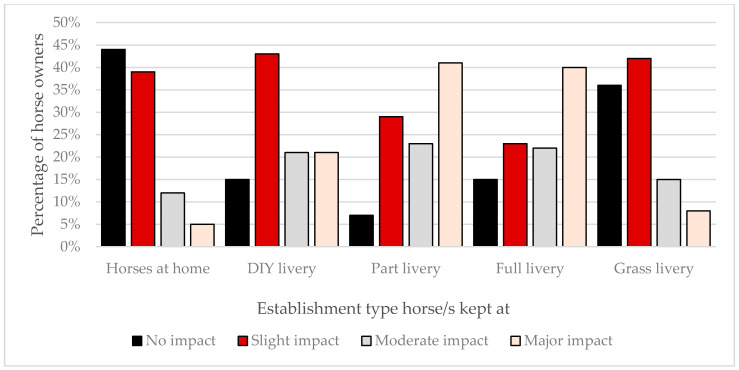
Impact of the coronavirus (SARS-CoV-2) pandemic on the management of United Kingdom (UK) horses by establishment type. Horse owners were asked to identify their perception of how much the COVID-19 pandemic had affected their normal day-to-day management of their horse/s. The data are grouped by the type of establishment.

**Figure 3:**
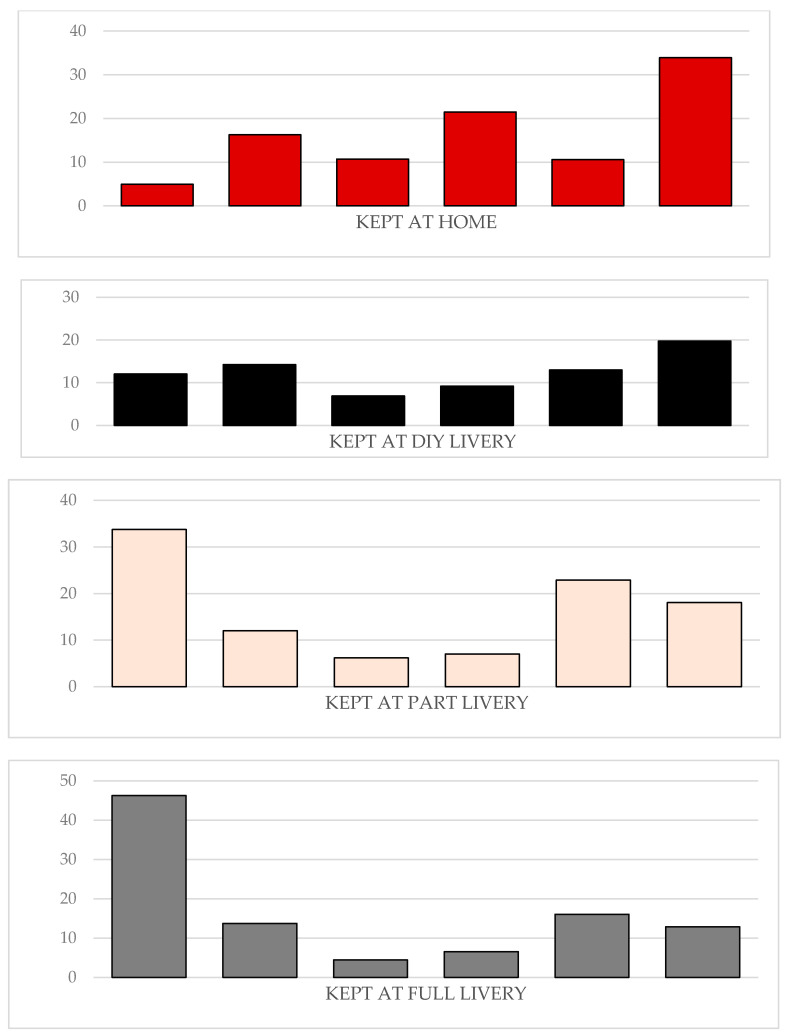

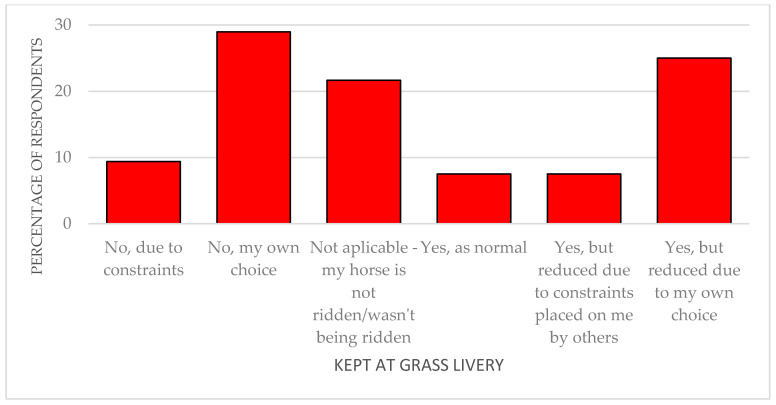
Differences in riding practices by establishment type. Horse owners were asked to select the option that best described how they were riding their horse since restrictions were implemented in response to the COVID-19 pandemic. The data are reported by establishment type.

The majority of UK horse owners surveyed stated that how they managed their horses had been affected by the current coronavirus pandemic. Owners who kept their horses within private livery yards reported they had experienced a greater impact from COVID-19 on the management of their horse/s (range: 65–93%) than owners who kept their horses at home (56%; Figure 2). The majority of management changes implemented were driven by the establishment and not the horse owner: full livery: 75%, part livery: 75%, DIY livery: 58%, and grass livery: 54%. Most owners with horses kept at full or part livery had experienced a reduction in the number of times they could visit their horse daily (79% and 78%, respectively). A reduction in daily visiting was also observed at DIY and grass livery establishments but at a reduced frequency (47% and 30%, respectively). Many respondents were still riding their horse/s (kept at home: 66%; full livery: 36%; part livery: 48%; DIY livery: 42%; and grass livery: 41%); however, the majority had adapted their riding in response to the pandemic (Figure 3). Few owners were concerned that restrictions related to COVID-19 would result in their horse/s having to be confined to their stable for extended periods (mean ± sd: 6 ± 0.05%). Approximately half of the owners who kept their horse at home (51%) or on DIY (50%) or grass livery (51%) had roughed off their horse because of the coronavirus pandemic; this figure was reduced for horses kept on part (41%) and full (44%) livery. The respondents generally had not made changes to their horse’s medical management: kept at home: 8%; full livery: 33%; part livery: 34%; DIY livery: 23%; and grass livery: 6%.

The majority of DIY, and part or full livery establishments had implemented social distancing practices on their yards (80%, 90%, and 89%, respectively); however, this was reduced to 21% when the horses were kept at grass livery and only 4% when the horses were housed at home. The respondents reported that hand washing facilities (range: 9% to 53%) and yards instigating allocated times for owners to visit horses (6% to 48%) were the most common practices put in place to reduce the spread of SARS-CoV-2. However, few establishments had implemented additional recommended protocols to prevent cross-contamination (Table 1).

**Figure 4:**
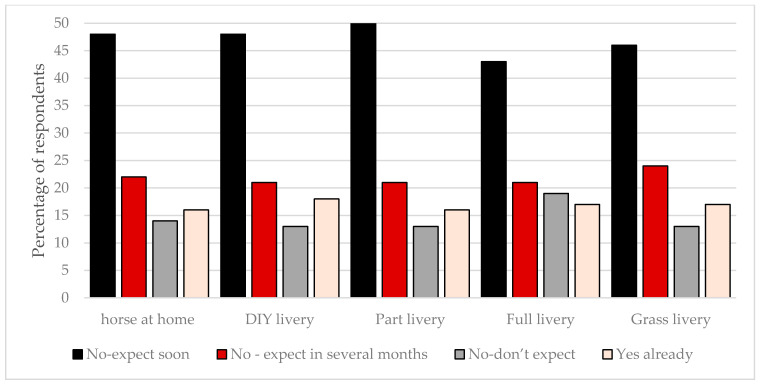
The extent to which changes to professional services for horses e.g., veterinarian, farrier, equine dentist, and physiotherapist, related to COVID-19 affected horse health. Respondents were asked to select the option that they felt best fitted their expectations for how the COVID-19 pandemic would affect their access to common professional services and subsequently their horse/s health. The data are reported by establishment type.

Horse owners who kept their horses at DIY, and part and full livery were more concerned that the restrictions associated with COVID-19 would affect their horse’s health (55%, 60%, and 62%, respectively) than those whose horses lived at home (35%) or at grass livery (9%). Whilst the majority of horse owners felt that changes to professional services, such as veterinarians, had not affected the health of their horse to date, most felt there would be some impact in the coming months (Figure 4). Just over a third of horse respondents (37%) owned a horse that required some form of medical management for conditions, such as laminitis, gastric ulcers, chronic lameness, or equine asthma. Few owners who kept their horses at home (8%) or at grass livery (6%) stated they were worried about their ability to care for their horses currently. However, changes to how owners managed their horses with medical issues had already occurred in 23% of horses on DIY livery, 34% on part livery, and 33% on full livery.

**Figure 5:**
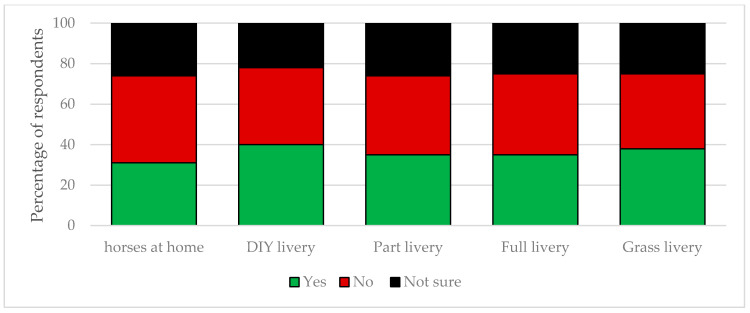
Percentages of horse owners who were concerned that the ongoing impacts of COVID-19 would make it difficult for them to provide for their horse’s essential needs. Respondents were asked to state if they thought the impact of COVID-19 pandemic would affect their ability to provide their horse/s essential management exercise and health requirements, if it would not affect this, or if they were not sure at this stage if there would be an impact. The data are reported by establishment type.

Approximately a third of horse owners were worried that the ongoing impacts of COVID-19 would make it difficult for them to provide for their horse’s essential needs, including livery costs, feed, forage, farrier, and veterinary bills. Whilst an additional quarter were not sure if their ability to care for their horse would be affected at some stage (Figure 5).

None of the changes reported to horse management differed significantly between livery establishments (*p* > 0.05).

### 3.2. Impact of COVID-19 by UK Region

The impact of the coronavirus (SARS-CoV-2) pandemic on horse owners was also evaluated across the different UK regions. No regional differences between the type of establishment were found (*p* > 0.05); however, the impact of coronavirus varied significantly in different parts of the UK (*p* < 0.05).

**Figure 6:**
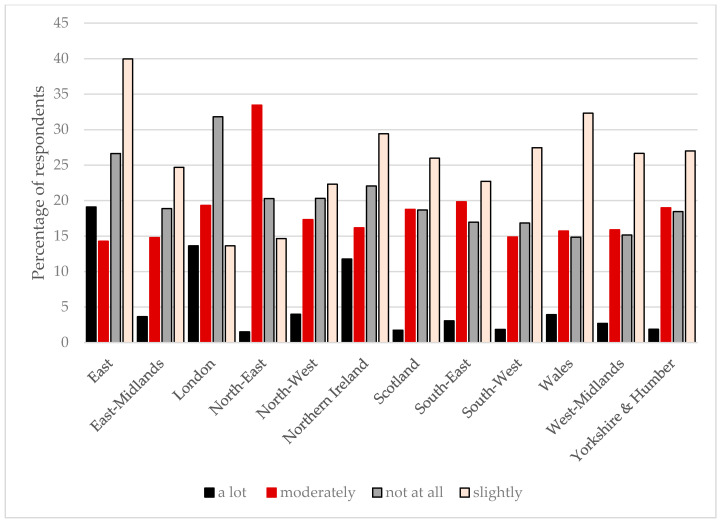
The impact of COVID-19 on horse owners by UK region. Respondents were asked to rank the impact of COVID-19 to date on the management of their horses and whether this had been affected a lot, moderately, slightly, or not at all. The data are reported by United Kingdom geographical region.

Owners in London, the East, and North East reported being the most affected by changes related to COVID-19 to date, with 33%, 33%, and 35%, respectively, of owners in these areas experiencing moderate or severe impacts (*p* = 0.0005; Figure 6).

**Figure 7:**
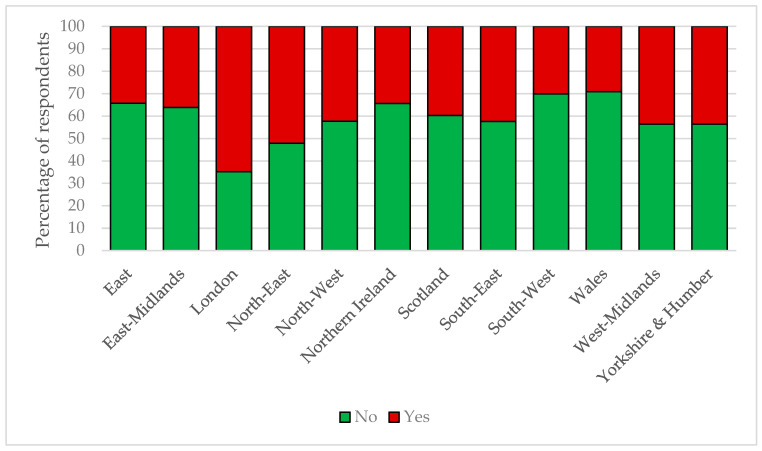
Percentages of horse owners experiencing a reduced frequency of visits to their horses due to COVID-19 restrictions across UK regions. Horse owners stated if they had or had not been required to visit their horse/s less since COVID-19 restrictions were implemented. The data are reported by United Kingdom geographical region.

Changes to equine management regimes were experienced across all regions, the majority of these had been self-imposed by the horse owner (London: 32%; North-East, Yorkshire and Humber and Northern Ireland range: 42–47%; and range in remaining regions: 52–60%; *p* = 0.0005). Significant reductions in how many times a day horse owners could visit their horse were found between the regions (*p* = 0.0005); London and the North-East were the most affected, while the South West and Wales were the least affected (Figure 7).

Significant differences were found in the choices horse owners were making regarding riding or not, by region (*p* = 0.0005). Increased numbers of horse owners in London and the North-East were not riding (45% compared to 30–37% in other regions), while across all regions ~40% of respondents were still riding, 13% ± 3% as normal and 41% ± 3% at a reduced frequency or with constraints on the type of activities undertaken (personal choice: 25% ± 4%; imposed: 16% ± 3%). Across the UK, horse owners in different regions were concerned that the impact of the coronavirus pandemic would result in changes to their access to professional services (*p* = 0.0005). To date, 17% ± 2% of horse owners had already experienced changes to these services and a further 67% ± 5% had not been affected currently but expected to experience issues within the next few months. Approximately 25% of horse owners were worried that the ongoing impacts of COVID-19 would make it difficult for them to provide for their horse’s essential needs. Horse owners in London (18%) were less concerned than horse owners from the other UK regions (24% ± 2%; *p* = 0.0005).

### 3.3. Horse Owner Perceptions of Current and Future Impact of COVID-19

**Figure 8:**
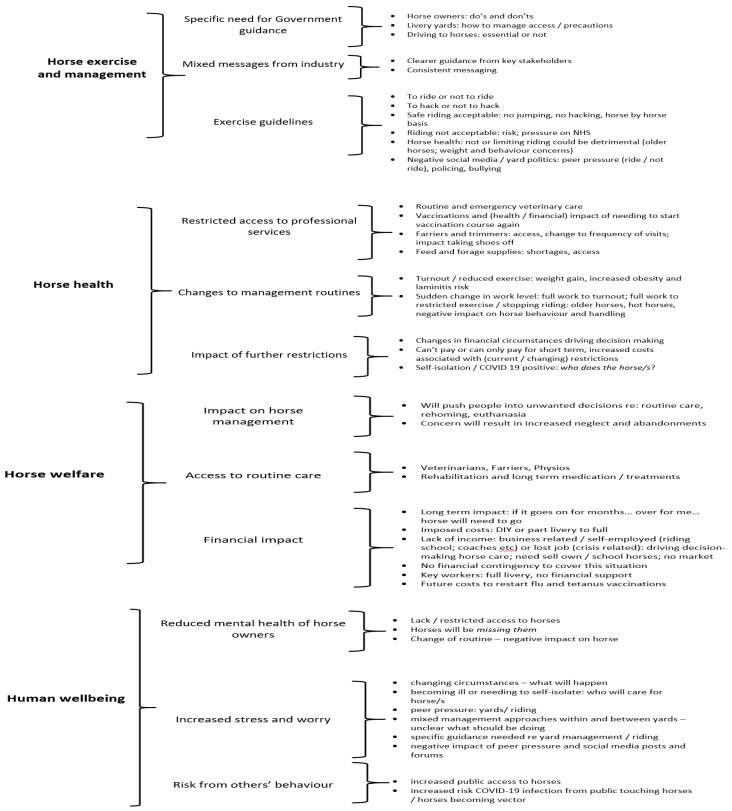
Thematic analysis of the horse owners’ perceptions of the current impact of the COVID-19 pandemic. Thematic analysis using a grounded theory approach identified themes from respondent’s answers to the open question: do you have any further comments on how COVID-19 has impacted on horses and horse owners. Four key areas of impact emerged: (1) horse exercise and management, (2) horse health, (3) horse welfare, and (4) human wellbeing.

Thematic analysis identified four higher order themes that respondents felt were related to the current and potential future impact of COVID-19 on the equestrian sector: horse training and management, horse health, horse welfare, and human wellbeing (Figure 8).

#### 3.3.1. Horses’ Health, Training and Management, and Welfare

The horse owners surveyed were concerned that changes made to their horses training and management regimes as a result of COVID-19 had the potential to negatively impact their horses’ health and welfare though reduced (owner) access and variation in exercise levels leading to weight gain and health issues in older horses: *“I own a highland pony who is on a strict diet and usually worked regularly to manage his weight. I have continued to ride as I worry turning him away would be detrimental to his health”*.

Generally, horse owners felt there had been mixed messages at the local, national, and Government level as to when they should or should not be riding or visiting their horse, and regarding what control messages should be implemented in equestrian facilities: *“Would like to ride, but feel I can’t, due to if I hurt myself taking up emergency services time”.* This confusion had resulted in anxiety and worry, as well as polarised peer pressure to conform to different perspectives on social media and at a local yard level: *“Horse needs exercise but feel pressured to not ride”, “There has been a lot of ‘advice’/abuse on social media towards people who carry on riding their horse as usual. Riding shaming”, “people need to stop online bullying towards people making different choices to themselves”*.

The provision of consistent and clear guidance from key industry stakeholders and yard management would be welcomed to help resolve these issues: “*Struggled to obtain guidance on daily visitation to tend to horses, had to rely on interpretations from various equine organizations”, “one person on the yard is still riding whilst all others have chosen not to… would like clear guidance from government about riding of horses used for personal use/hacking etc.”*.

As well as being concerned over the impact of the pandemic on their own horses, respondents articulated a wider unease regarding the broader impact of COVID-19 across the equestrian sector. These worries were predominately focused on the financial implications of the pandemic on individual horse owners and equestrian businesses, and equestrian charities: *“Cannot sleep for worry about paying for my horse. My employers have already cut my salary and today warned that redundancies will happen soon due to Coronavirus. Getting really depressed about it as my horse is my life and best friend”, “I have already lost my income so extremely worried about the immediate future”*.

Key fears were that a reduction in income would influence decision-making in horse owners and businesses potentially compromising horse welfare as owners could not afford to manage horses optimally, or could result in increased relinquishments, leading to increased pressure and burden on equestrian charities: *“I had to have my mare put to sleep as she has been very poorly and I could not take the risk of the vet not being able to attend. I’m heartbroken”, “Very concerned about welfare cases, think we will see more”, “I am concerned that many people will have to give up their horses as they can’t afford them which will flood rescue charities.”.* Limited access to veterinary and other professional services was also a concern for horse owners, who felt that changes in how they accessed these services could negatively impact their horses’ health and welfare: “*My main concerns are: Getting ill at the same time as those who could cover for me to care for all the animals. Loss of income making it harder to provide for emergencies. Vet and farrier availability, when needed*”, “*Our yard has banned visits, very harsh. Also worried about getting horse shod properly as farrier only undertaking urgent work”.*

#### 3.3.2. Human Wellbeing

Alongside concerns for the health and welfare of their horses, horse owners also strongly felt they were personally experiencing a negative impact of the pandemic on their mental health and wellbeing. This was occurring as a result of increased stress and worry related to the pandemic and how they would adapt to future changes that could affect their horses’ management, if they became ill with the virus, coping with the uncertainty within the current situation and not having access to their horse as much as they would like to, which for many had been a key coping strategy to reduce anxiety pre-COVID-19 restrictions: “*As a widow living on my own, I really miss the time with my horse. It was time spent riding/with him that cheered me up*”, “*It has had an effect on my mental health as well as others I know not being able to spend as much time with our horses in fresh air and being confined indoors because of lock down*”, “*My pony is my sanity*”, “*I miss my horses!”*, *“No one seems to be concerned on horses or owners mental health…My horses are my life as well as my daughters, they are not field ornaments and are used to some sort of work”.*

## 4. Discussion

Our results have recorded a snapshot of the initial impact of the coronavirus (SARS-CoV-2) pandemic on UK horse owners. The pandemic is influencing how owners interact with their horses across all UK regions and all types of livery establishments. The greatest impact to date has occurred in virus hot-spots: London and the South-East, and for owners who keep their horses at part and full livery establishments, where increased visiting and management restrictions were reported.

### 4.1. Management of Impact of COVID-19 within Equestrian Facilities

The rapid implementation of the quarantine measures to control the impact of COVID-19 introduced in the UK had a significant and immediate effect on the equestrian sector. Restrictions regarding the access to horses due to COVID-19 initiated sudden changes within their management, resulting in many fit horses being roughed off to pasture or increasing their time at pasture in response. The management regimes of competition and recreational horses are routinely subject to change related to seasonal, geographic, breed, age, and discipline/use factors, as well as in response to their owners’ financial circumstances [30,31]. In the UK, the majority of horses are stabled with some access to turnout; however, the amount of time at pasture varies with accessibility, seasonality, and with competition schedules and weather [30,31,32,33].

Short term reductions in exercise or ‘holiday’ periods are often integrated into competition horse schedules and are not considered detrimental to equine health or welfare if accompanied by appropriate management changes, including a reduction in calorie intake to match the revised exercise levels. Short term breaks are often anecdotally reported to exert a beneficial impact on horses’ psychological and physical wellbeing. Therefore, although owners expressed their worries that management changes made in response to quarantine restrictions would negatively affect their horses, if the transition is managed effectively, this should not be the case. However, a recent survey [34] identified a lack of fundamental knowledge relating to equine health and welfare, and examples of poor knowledge or owner or rider ignorance contributing to reduced welfare are well documented within recreational and competitive equine populations [18,35,36].

Whilst industry guidelines exist for certain aspects of management, such as feeding protocols and stable size, their application is limited within the wider equestrian practice, where many accepted practices are predominately based on anecdotal and historic methods [18,37]. Based on our results, we would recommend key influencers in the equestrian sector, including the national federation, British Equestrian, and its member bodies work in partnership with equestrian charities to engage in increased communication and education strategies that support horse owners to manage their horses effectively through the changing phases of the pandemic.

Swift changes in management regimes, such as roughing off at short notice or reduced visits potentially resulting in condensed management of horses in response to the COVID-19 restrictions, can also represent risk factors for equine disease. Horses kept predominately at pasture have increased access to forage with a higher water content (subject to the condition of the grazing available) than stabled horses and need to move more to source their feed, spending 55% of their time eating at pasture compared to 15% when stabled [30,38]. Quarantine restrictions were introduced in the UK in March, coinciding with climate conditions that would generate new grass growth and the highest levels of sunshine on record for April [39].

Laminitis is a highly debilitating disease of the horse’s foot associated with relentless pain and degenerative changes, which has major equine welfare implications and can often necessitate euthanasia on welfare grounds [40,41]. An increased risk of laminitis has been associated with reduced exercise levels, increased hours of sunshine [42], and increasing age in horses [40]. Wylie and colleagues [40] found horses with new access to grass within the preceding four weeks were seven times more likely to have laminitis compared to those with access for longer than four weeks or no prior access, and owner reported weight increases in horses within the previous three months were associated with a four-fold increase in laminitis [40]. Research also documented that horse owners cannot always easily recognise the clinical signs of laminitis [43] and this, combined with the 30–79% reduced access to horses due to the imposed restrictions reported by respondents, could result in owners not identifying the onset of laminitis as readily.

Between half and two-thirds of owners surveyed had experienced management changes as a result of the coronavirus pandemic, with, on average, 36% also reducing how often they were riding their horses. Recent changes in management regimes related to exercise and stabling were associated with an increased incidence of colic in horses [31,44]. Risk factors reported included having more than three carers looking after a horse [45], a change in housing or turnout within two weeks [45,46], increased hours stabled in the preceding two weeks [47], change in pasture during the last 28 days [47], and a recent change in the exercise routine [46,48], all of which could have occurred as a consequence of the restrictions associated with COVID-19. Horse owners highlighted their concerns that the increased need for horses to be cared for by third parties who lack familiarity with their horse could negatively impact the horse health and welfare.

We would advise that horse owners and other carers are particular vigilant in monitoring for the clinical signs of colic during periods of change and transitions in equine management and exercise regimes. Moving forward, as quarantine restrictions begin to reduce, owners should also consider the potential of associated management changes (e.g., increasing workloads and a return to increased time spent stabled) to their horses’ health. Williams et al. [31] reported that horses increased their water intake, reduced their faecal output, but with an increase in faecal dry matter content, and had reduced colon motility when moving from pasture to stabling with light exercise for a period of five days. Therefore, horse owners and carers should implement a gradual transition back to previous levels of work and stabling, whilst monitoring clinical signs that could indicate the onset of any health issues.

The fitness levels of horses should also be considered when returning them to work. Short rest periods at pasture (~2 to 3 weeks) can have a beneficial impact on horses’ psychological and physical wellbeing; however, extended periods of turnout or decreased exercise levels will reduce fitness. Equestrian practice is multifaceted and, to achieve optimum management, due consideration of the appropriate nutrition, housing, behaviour, and riding and training practices, as well as ensuring that the horse is sufficiently prepared in terms of fitness and skill development to meet the demands of the workload or competition expected is required [49]. Horse owners should consider this once horses are brought back into work and introduce a gradual fittening programme, alongside feeding for the level of work being undertaken to prevent unwanted behavioural responses that could compromise the horse and rider safety.

### 4.2. Long Term Implications of the Pandemic on Equine Welfare

Reduced access to veterinary and other professional services was a key concern of the horse owners surveyed. At the time of writing, equine veterinarians are undertaking emergency visits only and access to farriers, equine dentists, and equine physiotherapists is limited. Horse owners and trainers are responsible for the management of their horses and have a duty of care to engage in practices that optimise equine health and welfare [18,19]. The current restrictions to veterinary and related services has the potential to prevent owners executing this duty of care and to compromise equine welfare as a result of circumstances beyond their control. The horse owners surveyed here were particularly concerned regarding the future potential impact of equine influenza as a result of horses not being able to have booster vaccinations due to COVID-19 restrictions.

The long term financial impact of COVID-19 was also a key concern for the majority of respondents. A third of horse owners stated they were worried they would experience a negative financial impact of the pandemic, which could affect the management decisions they make for their horse. Broader concerns for the financial impact on the wider equestrian sector and particularly equine charities were also expressed if restrictions associated with the pandemic occur for extended periods impacting personal finances.

The UK equestrian sector is considered to be experiencing an ongoing equine welfare crisis since the start of the current decade. The result is an increasing burden on equine charities, which, despite often already operating close to capacity, have adapted their practice to provide support for welfare cases, create additional homes for unwanted horses, and provide education for horse owners [50]. Many equine charities rely on volunteers, fundraising activities, social events, and engagement with the general public, activities that are generally prohibited under the quarantine restrictions. This, combined with a potential reduction in donations due to the wider economic impact on supporters, will place more pressure on charities at a time when their services may become more in demand to protect equine welfare.

The size and persistence of the economic impact of the COVID-19 pandemic is unknown. A short-sharp crisis is looking less likely with experts predicting associated national and global recessions as a result of the pandemic [51,52]. The disease is still spreading, and it is likely that social distancing measures will be imposed, nationally and globally, for between 12 and 18 months, or until a vaccine is developed to avoid severe public health consequences [53]. These restrictions, combined with an increased burden on medical services associated with increased admissions due to COVID-19, will impact individuals, particularly those employed in sectors revolving around leisure, tourism and sport for a significant period of time [27,53].

The broader influence of restrictions resonates throughout the equestrian sector. Slowing down the spread of COVID-19 to manageable levels for the health systems to operate has to remain the number one priority for the UK; social and sporting gatherings present a genuine risk to potentiate the transmission of the virus [27]. Restrictions are affecting all aspects of equestrianism, for example competitive equestrian events have been postponed or cancelled, riding schools are restricted in what services they can offer to clients, and recreational riding is severely limited.

Whilst it is likely that much of the 2020 competitive season may be lost to COVID-19 across many equestrian sports, some aspects of the industry, such as racing, are exploring alternative options including running race meetings behind closed doors. The economic consequences of these changes, postponements, and cancellations are as of yet unknown, but many commentators and those within the sport expect this to fundamentally change the way sport operates in the future [27]. For equestrianism, ancillary industries, such as the breeding and bloodstock sector, are also likely to be severely impacted.

### 4.3. Horse Owner Well-Being

Across all UK regions and types of establishment, horse owners felt that the restricted access to their horses and what activities they could do with them was having a detrimental impact on their mental health and wellbeing. Despite a lack of empirical evidence regarding the positive effect of the human–animal bond on human wellbeing, there is a commonly held belief that companion animal attachment (rather than ownership) contributes to improved wellbeing [54,55]. Interaction with companion animals was reported to reduce feelings of loneliness and depression [56,57], reduce levels of stress [58], reduce anxiety [59], improve feelings of self-worth and self-esteem [60], and increase emotional and social support [61,62]. Interactions with a familiar animal appear to potentiate the positive effects on wellbeing [55]. A close familial bond was embedded across survey responses.

A shared sense of co-being between horse and rider alongside horse owners describing their equine partners as soul mates commonly defines horse–human relationships [22], and it is this bond that expedites positive influences on human wellbeing when interacting with a familiar horse [23,24,63]. The sudden restriction on access to horses and the green spaces they commonly inhabit, may have not only limited the physical exercise of horse owners, but concurrently contributed to increased levels of anxiety and loneliness at a time when external pressures and quarantine measures related to COVID-19 were likely to already be amplifying these feelings. Some form of social distancing restrictions are likely to remain in place until a vaccine for COVID-19 is available [53]; therefore, strong biosecurity protocols may be a key way forward to support increased contact between horses and their owners. Our results found that the majority of equestrian establishments had implemented social distancing measures but that wider controls, such as the provision of hand and equipment sanitisation facilities combined with targeted visiting protocols, could facilitate increased access to horses and support owner mental wellbeing.

### 4.4. Limitations

The current study did have some limitations. Participation was voluntary and, as a result, the results may be subject to inherent subject and response bias and could over represent the views of horse owners who have been impacted more by the coronavirus pandemic and those who engage with online platforms. The nature of the survey tool utilised could allow the same individual to complete the survey more than once, which has the potential to introduce sampling bias. The engagement of social media sites and third parties to support the distribution and advertising of the survey could have resulted in some degree of cultural or conformation bias if the respondents were affiliated to the specific sites or organisations utilised. Specific reports of colic or laminitis were not reported by owners in the survey, and, while potential associations to these conditions as a result of management changes related to quarantine restrictions were discussed, it is beyond the scope of this survey to directly assess the occurrence of these conditions as a result of COVID-19-induced management changes

## 5. Conclusions

The coronavirus (SARS-CoV-2) disease outbreak and subsequent government quarantine restrictions have had a major impact on the management of leisure and competition horses, and their owners within the United Kingdom. Horse owners who keep their horse in private livery establishments have experienced more changes to their own and their horses’ normal management regimes and riding less than owners who keep their horses at home. Most livery yards have implemented social distancing measures to reduce the risk of cross-contamination but nearly half of those surveyed had not been provided with hand washing/sanitization facilities and did not have to follow any restricted visiting or disinfection protocols at their livery yards.

Horse owners expressed their concerns at the ongoing impact of the coronavirus pandemic on the health and welfare of their own horses and the general UK equestrian sector. Owners felt that the reduced access to professionals, such as veterinarians, equine dentists, equine physiotherapists, and farriers, could be detrimental to equine health. The potential negative financial impact of COVID-19 was a key anxiety, with owners feeling that they or others may have to make decisions concerning horse management based on affordability, which could lead to increased relinquishments and additional burdens on equine charities, and also result in negative equine well-being. The impact of the pandemic on human health was also apparent with horse owners consistently articulating the impact of not being able to visit and interact with their horse(s) as much as they typically would negatively affecting their mental health and wellbeing.

It is unlikely that all quarantine and social distancing measures related to COVID-19 will be removed until a vaccine for SARS-CoV-2 is available; therefore, whilst access restrictions have reduced at the time of writing for many horse owners in the UK, the impact of the coronavirus pandemic will be felt for the foreseeable future. There is a need for key influencers in the equestrian sector to work with equestrian charities to implement increased communication and education strategies to support horse owners to manage their horses effectively through the changing phases of the pandemic and protect both human and equine health and welfare.

## Figures and Tables

**Table 1:** Frequency of measures implemented in response to the coronavirus (SARS-CoV-2) pandemic across establishment types. Horse owners were asked to confirm if the establishment they kept their horse/s within in had provided the commonly recommended measures to reduce the cross-contamination of SARS-CoV-2 between humans. The data are reported as the sum number of establishments that did provide the measure.

Establishment	Home	DIY Livery	Part Livery	Full Livery	Grass Livery
Restricted to one visit per day	3%	2%	41%	29%	8%
Set up ‘buddy’ or peer group to manage horses	2%	27%	12%	8%	7%
Provision of hand sanitiser or soap	11%	45%	53%	48%	9%
Disinfection protocols and facilities provided for shared areas and equipment	7%	25%	32%	30%	5%
Allocated times to visit horses	6%	34%	48%	39%	9%
Formal advice or guidance provided regarding COVID-19	4%	32%	36%	31%	4%
No measures put in place	NA	10%	1%	2%	5%

## Supplementary Materials

The following are available online at https://www.mdpi.com/2076-2615/10/10/1862/s1, Supplementary file S1: Effect of Coronavirus Pandemic on United Kingdom Horses and Horse Owners Survey Questions.
