# Supplementary material for: COVID-19: Impact on United Kingdom Horse Owners
Source: Animals (Basel). 2020 Oct 13;10(10):1862. doi: 10.3390/ani10101862 (PMC7600939; doi:10.3390/ani10101862)
Supplement: Supplementary file 1 [file animals-10-01862-s001.pdf]

Supplementary File 1:

Effect of Coronavirus Pandemic on United Kingdom Horses and Horse Owners Survey Questions

The survey was designed as an online questionnaire (Survey Monkey®, San Mateo, CA, USA) with 16 closed questions and 1 open free text question. Details of question text and available responses are provided in Table S1 below.

Table S1: Survey questions overview; MCQ: multiple choice question

Question number	Question type	Question text	Answer choices
1	MCQ	Which area of the UK are you in?	South West<br>South East<br>London<br>East<br>East Midlands<br>West Midlands<br>Wales<br>North West<br>Yorkshire and Humber<br>North East<br>Scotland<br>Northern Ireland
2	MCQ	How many horses and or ponies do you have?	1<br>2<br>3<br>4<br>5-10<br>More than 10
3	MCQ	Do you keep your horses/ponies....	At Home - Stables and/or Field<br>At a Livery Yard - DIY<br>At A Livery Yard - Part Livery<br>At a Livery Yard - Full Livery<br>In a Field (not your own)<br>Other (please specify)
4	MCQ	Have you had to change the management of your horse because of the COVID-19 (Coronavirus) pandemic?	Not at all<br>Slightly<br>Moderately<br>A lot
5	MCQ	If you have had to make changes were these your own choice?	Yes<br>No
6	MCQ	Have you had to reduce the number of times a day you visit your horse/horses?	Yes<br>No
7	MCQ	Are you still able to ride your horse?	Yes, as normal<br>Yes, but reduced due to constraints placed on me by others<br>Yes, but reduced due to my own choice<br>No, due to constraints<br>No, my own choice

			Not applicable - my horse is not ridden/wasn't being ridden
8	MCQ	Have you been told or seen advice to "rough your horse off" and turn him/her away to the field?	Yes<br>No
9	MCQ	Is your horse likely to have to undergo prolonged stable confinement as a result of COVID-19?	Yes<br>No<br>Not sure
10	MCQ	If your horse has medical/health issues that require specific management (e.g. EMS, laminitis, gastric ulcers, chronic lameness, asthma, sweet itch, etc) has your yard accommodated for horses' needs?	Yes - I don't have to follow rules set by yard manager/owner<br>Yes - I have had to change my management but appropriate measures have been put in place to ensure the health<br>of my horse<br>No - I have no control over the future management of my horse<br>Not applicable - my horse does not have any medical issues<br>Not applicable - my horse lives at home
11	MCQ	In light of the changes to routine needed to reduce the spread of COVID-19, are you concerned for the health of your horse?	Yes<br>Slightly<br>No
12	MCQ	Has your yard implemented measures to allow for social distancing?	Yes<br>No<br>Not applicable - I keep my horse privately
13	MCQ	Do you feel the measures which have been implemented at your yard are satisfactory?	Yes<br>No<br>Not applicable - I keep my horse privately
14	Checkbox – multi answer	Please indicate which measures have been implemented at your yard (please tick all that apply)	One visit to horse per day<br>"Buddy" group set up<br>Hand sanitisers or soap and water made available<br>Disinfection of shared areas or items (e.g. brooms, wheelbarrows, forks)<br>Allocated visiting times to avoid contact with others<br>Formal advice/instructions on COVID-19 printed and posted or sent by email/mobile on policy<br>No measures have been implemented<br>Not applicable - I keep my horses privately<br>Other (please specify)

15	MCQ	Have changes to services provided by equine professionals such as vets, farriers, dentists and physios affected the health of your horses?	Yes<br>No - But expect they will at some stage soon<br>No - But expect they will in several months' time<br>No - Don't expect them to at anytime
16	MCQ	Many people have lost or may lose their jobs/income as a result of COVID-19. Are you concerned that the ongoing situation may make it difficult to provide for your horses' needs (e.g. livery costs, feed, forage, farrier bills, vets bills)?	Yes<br>No<br>Not sure<br>Prefer not to say
17	Open	Are there any other comments you would like to make?	Open response
